# Supplementary material for: Investigating speech and language impairments in delirium: A preliminary case-control study
Source: PLoS One. 2018 Nov 26;13(11):e0207527. doi: 10.1371/journal.pone.0207527 (PMC6261049; doi:10.1371/journal.pone.0207527)
Supplement: S1 Table — IQR = Inter-Quartile Range. Variables derived from the Cookie theft picture description task are denoted with a *. Kruskal-Wallis and Mann-Whitney U were used. (DOCX) [file pone.0207527.s002.docx]

Table S1. Descriptive statistics and statistical group comparisons (Kruskal-Wallis and Mann-Whitney U) for scores on the language assessment.

IQR = Inter-Quartile Range. Variables derived from the Cookie theft picture description task are denoted with a *.

|  | **Delirium** | **Dementia** | **Cognitively unimpaired** | **Statistical test results** | **Pairwise comparisons** |
| --- | --- | --- | --- | --- | --- |
| **Language production** | Median (IQR) | Median (IQR) | Median (IQR) |  |  |
| Conversational speech: relevant content (range 0-9) | 7 (5 - 8) | 7 (5 - 8) | 9 (8 - 9) | H = 9.57, p <0.01 | Del vs Con: U = 52.50, p < 0.01  Del vs Dem: U = 107.00, p = 0.82  Dem vs Con: U = 48.00, p < 0.01 |
| Number of words per utterance* | 4.2 (3 - 7) | 7.5 (5.43 - 9.33) | 8.7 (6.33 - 11.82) | H = 11.62, p < 0.01 | Del vs Con: U = 40.50, p <0.01  Del vs Dem: U = 47.00, p < 0.01  Dem vs Con: U = 86.50, p = 0.28 |
| Content score* | 4 (0 - 5) | 6 (3 - 7) | 12 (9 - 14) | H = 17.74, p < 0.001 | Del vs Con: U = 23.50, p <0.001  Del vs Dem: U = 78.00, p = 0.15  Dem vs Con: U = 33.00, p < 0.01 |
| Fluency score* | 6.74 (1.43 - 14.13) | 3.89 (2.59 - 6.67) | 2.95 (0 - 6.19) | H = 3.60, p = 0.17 |  |
| Grammar score* | 1.09 (0 - 4.28) | 1.96 (0 - 2.7) | 0 (0 - 1.61) | H = 1.26, p = 0.53 |  |
| Semantic score* | 0 (0 - 0.59) | 0 (0 - 1.72) | 0 (0 - 0.77) | H = 0.88, p = 0.64 |  |
| **Language comprehension** |  |  |  |  |  |
| Verbal (range 0-4) | 4 (3 - 4) | 4 (4 - 4) | 4 (4 - 4) | H = 7.57, p = 0.02 |  |
| Written (range 0-4) | 3 (0 - 4) | 4 (3 - 4) | 4 (4 - 4) | H = 12.54 p < 0.01 | Del vs Con: U = 67.5, p < 0.01  Del vs Dem: U = 74.50, p = 0.08  Dem vs Con: U = 82.50, p = 0.04 |
